# Efficient generation of a stable CHO-K1 cell line overexpressing the human water channel aquaporin-5 as tool to generate therapeutic antibodies

**DOI:** 10.1038/s41598-024-67147-x

**Published:** 2024-07-10

**Authors:** Lucas Jagemann, Nia Sciucca, Michele Bombardieri, Elisa Corsiero

**Affiliations:** Centre for Experimental Medicine & Rheumatology, William Harvey Research Institute, Barts and The London School of Medicine and Dentistry, Queen Mary University of London, John Vane Science Centre, Charterhouse Square, London, EC1M 6BQ UK

**Keywords:** Stable cell line, Membrane proteins, Water channel, Aquaporin-5, Biological techniques, Biological models, Immunological models

## Abstract

Aquaporins (AQPs) are a family of water permeable channels expressed on the plasma membrane with AQP5 being the major channel expressed in several human tissues including salivary and lacrimal glands. Anti-AQP5 autoantibodies have been observed in patients with Sjögren’s syndrome who are characterised by dryness of both salivary and lacrimal glands, and they have been implicated in the underlying mechanisms of glandular dysfunction. AQP5 is formed by six transmembrane helices linked with three extracellular and two intracellular loops. Develop antibodies against membrane protein extracellular loops can be a challenge due to the difficulty in maintaining these proteins as recombinant in their native form. Therefore, in this work we aimed to generate an efficient stable-transfected cell line overexpressing human AQP5 (CHO-K1/AQP5) to perform primarily cell-based phage display biopanning experiments to develop new potential recombinant antibodies targeting AQP5. We also showed that the new CHO-K1/AQP5 cell line can be used to study molecular mechanisms of AQP5 sub-cellular trafficking making these cells a useful tool for functional studies.

## Introduction

Aquaporins (AQP) are a family of transmembrane water permeable channels with AQP5 being the major water channel expressed in both human salivary (SG) and lacrimal glands with a critical role in tear and saliva secretion^1,2^. AQP5 is formed by six transmembrane helices linked with three extracellular and two intracellular loops. It consists of four monomers each of which contains a pore permeable to water. AQP5 encounters dynamic regulations including trafficking from the cytoplasm to the plasma membrane. This trafficking is important for the regulation of physiological properties, like water control. Thus, AQP5 localization is not only limited to the plasma membrane where it exerts its role, but it can be found in the cytoplasm as well. The C-terminal domain of AQP5 is implicated in the plasma membrane localization, i.e. upon stimulation with cAMP^3^.

The mechanism of salivary secretion in SG acinar cells is started by the release of acetylcholine by the parasympathetic nerves, with an increase in the intracellular Ca^2+^ level, which ends in the activation of the Ca^2+^-dependent K^+^ and Cl^−^ channels. Upon muscarinic M3 receptor stimulation, AQP5 moves from the cytoplasm to the apical membrane of the SG acinar cells, thus inducing fluid secretion in SG. Several authors have also showed that AQP5 translocation can be regulated by cAMP probably due to the presence of a PKA consensus sequence in one of its cytoplasmic loops^3,4^.

AQP5 has been associated with sicca symptoms in Sjögren’s syndrome (SS). SS is the second most common rheumatic autoimmune disorder (0.5–1% in the adult population) characterised by chronic inflammation of exocrine glands, particularly SG and lacrimal glands, leading to xerostomia/dry-mouth and keratoconjunctivitis/dry-eyes sicca symptoms. Thus, dryness is one of the major problems affecting the quality of life of SS patients. There is no cure that can induce remission and the therapeutic approaches aim toward symptoms palliation and prevention of complications, particularly the sicca symptoms. Artificial tears, saliva replacements or stimulation of saliva flow are the current treatments used for sicca symptoms in SS patients. These treatments provide short-term relief and require a continual use to reach maximal benefits. Recent evidence has shown both a problematic translocation of AQP5 on the SG cell membrane and the presence of circulating autoantibodies against AQP5 in SS patients which might induce functional impairment of the secretory process and salivary dysfunction^1,2,5,6^. Therefore, developing effective and targeted therapies that could restore the secretory process represents an important unmet need to combat the disease.

Antibody phage display technology has been proven for its safety and efficiency for developing specific monoclonal antibodies to use in clinical research. A phage displaying a specific antibody on its surface can be isolated based on its binding property to a specific target and the linkage between the antibody gene inside and protein displayed on the surface facilitate robust development of antibody candidates. An antibody library can be screened against target proteins to isolate antibodies of defined specificity, a process called biopanning. The successful development of monoclonal antibodies using phage display depends on the quality of the antigen that should be kept in its native conformation as much as possible. Methods to perform biopanning on soluble purified proteins are well established. For membrane proteins, like AQP5, which contain hydrophobic transmembrane and extracellular domains, maintaining the native form as recombinant protein is more difficult. The expression of membrane proteins in a soluble form is very challenging and difficult to achieve. To overcome this problem, one of the strategies has been to use whole cells as antigen source for biopanning to isolate antibodies biologically relevant to a specific epitope of the target antigen^7,8^.

To our knowledge, no commercially stable cell line expressing human AQP5 is available. Therefore, here we aimed to develop and characterise a stable cell line expressing human AQP5 that could be used to perform cell-based biopanning and potentially functional studies.

## Results

### Generation of stable CHO-K1/AQP5 cell line

**Figure 1:**
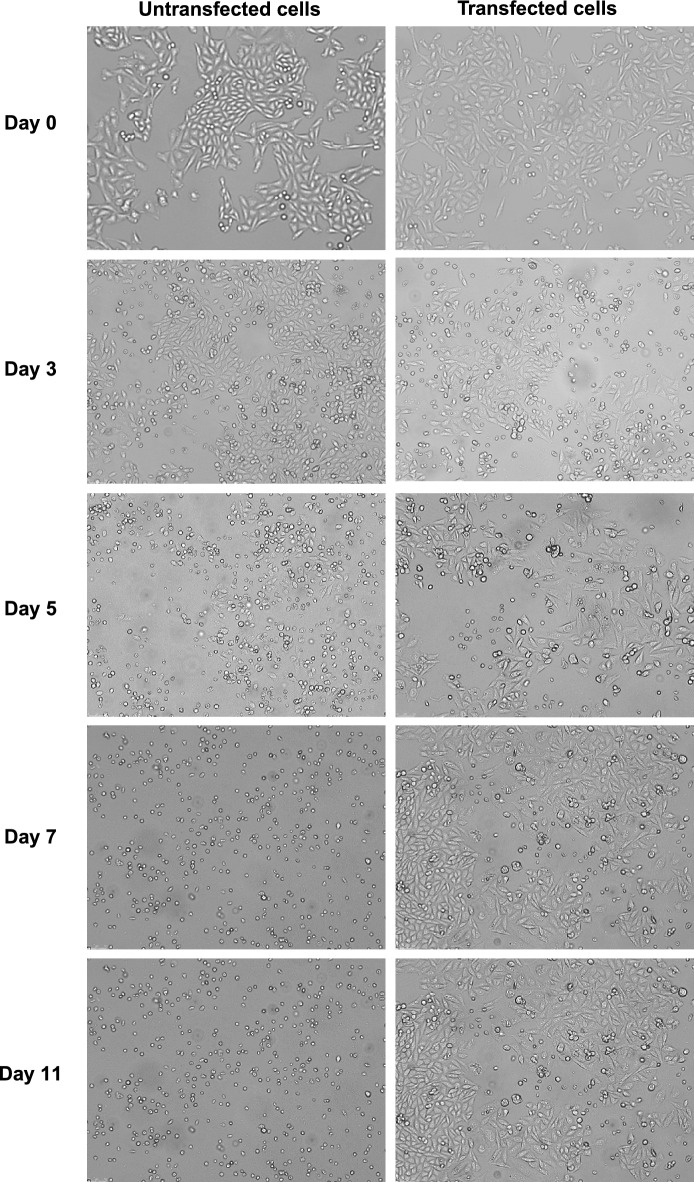
CHO-K1 transfection with hAQP5 plasmid. Representative bright field images of untransfected (left panel) vs transfected (right panel) CHO-K1 cells at day 0, day 3, day 5, day 7 and day 11 post-transfection with a human AQP5 plasmid. All images have been acquired using the inverted microscope. Magnification: ×20.

After transfection, stable-transfected CHO-K1 cells were selected by the addition of hygromycin B to the culture medium since the expression plasmid encoding for hAQP5 carries the hygromycin B as drug resistance gene. The hygromycin B concentration was determined using batch culture by choosing the concentration that was 0.1 mg/ml above the one showing complete cell death (data not shown). CHO-K1 cells which have not integrated the resistance gene normally die during the first days of selection. As shown in Fig. 1, the untransfected cells started dying after 5 days post-transfection. We kept the transfected CHO-K1 cells in culture under selection pressure up to 11 days where it was possible to observe the outgrown of resistant cells (Fig. 1).

**Figure 2:**
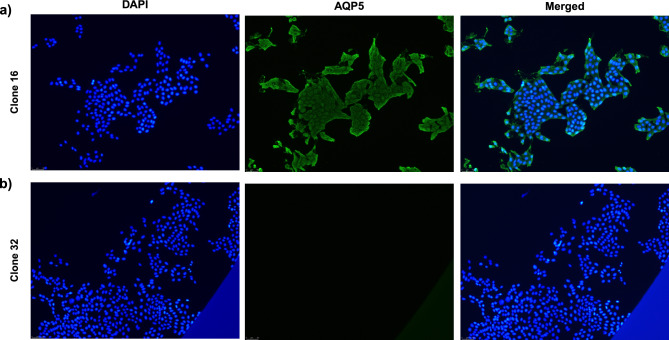
Characterization of CHO-K1/AQP5 clones. Representative immunofluorescence pictures showing staining for AQP5 (green) for clone 16 (**a**) and clone 32 (negative clone) (**b**). Nuclei were stained with DAPI (blue). All images have been acquired using the inverted fluorescence microscope. Magnification: ×20.

We then performed a clonal selection by diluting the batch culture to one cell/well to have a uniform stable-transfected cell line derived from one transfected cell (Supplementary Fig. S1). As shown in Fig. 2a and Supplementary Fig. S2, we selected one clone (clone 16) which showed a strong expression of AQP5 by immunofluorescence staining compared to a clone (clone 32) not expressing AQP5 (Fig. 2b). As expected, AQP5 showed both a membrane and cytoplasmic distribution.

**Figure 3:**
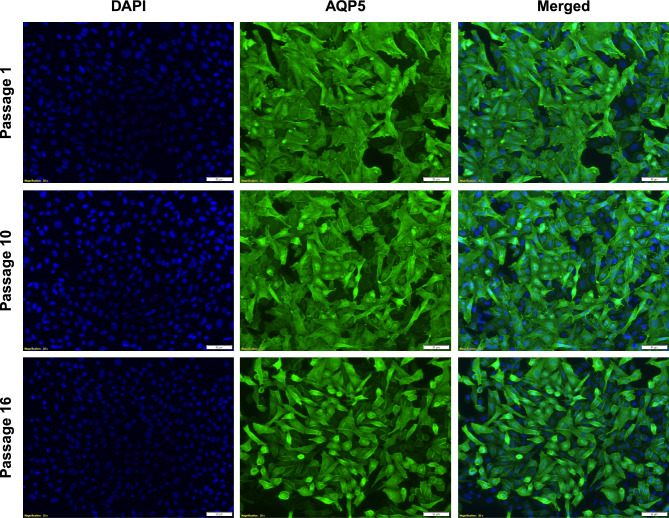
AQP5 expression stability over-time. Representative immunofluorescence pictures showing staining for AQP5 (green) for clone 16 at different time points indicated as passage 1, passage 2 and passage 16. Nuclei were stained with DAPI (blue). Magnification: ×20.

Finally, we followed the AQP5 expression over time in the clone 16 by immunofluorescence staining. We showed that after two weeks post-transfection (passage 16), the transfected cells were still able to express AQP5 at high level (Fig. 3).

### AQP5 detection at protein level in the CHO-K1/AQP5 cell line

**Figure 4:**
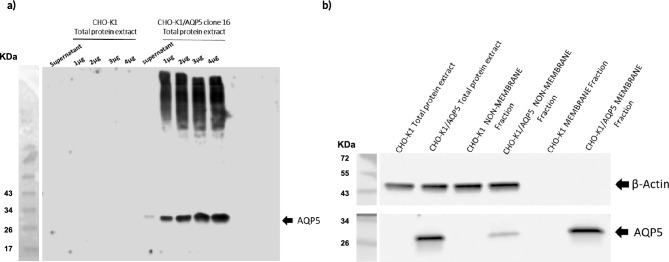
AQP5 protein expression and plasma membrane localization. (**a**) AQP5 expression on supernatant and total protein extract (1–4 µg) from CHO-K1 and CHO-K1/AQP5 cells (clone 16) assessed by western blot. (**b**) AQP5 expression on non-membrane and membrane fraction from CHO-K1 and CHO-K1/AQP5 cells (clone 16) assessed by western blot. β-actin was used as control.

To prove the AQP5 expression at protein level for the clone 16, we first performed a Western blot analysis on total protein extract obtained from the stable-transfected clone. The untransfected CHO-K1 cells did not shown any presence of AQP5 (Fig. 4a). The clone 16 showed a clear band around 28 KDa corresponding to AQP5 (Fig. 4a). We also observed a residual expression of AQP5 in the cell culture supernatant for the clone 16, probably derived from dying cells releasing the protein extracellularly and the presence of higher bands in the range of 100 KDa corresponding likely to tetramers, trimers or dimers form of AQP5 protein which are resistant to SDS^9^.

Next, we proved the presence of AQP5 on the cell plasma membrane. We performed a membrane fractionation by dividing the membrane proteins from the total protein extract. As shown in Fig. 4b, AQP5 expression was detected by Western blot mainly on the cell membrane with a residual expression in the non-membrane fraction. The untransfected CHO-K1 confirmed the absence of protein expression. The presence of the HIS-tag in the N-terminal of the AQP5 protein was also confirmed by ELISA assay using either an anti-HIS-HRP antibody or Nickel-HRP (Supplementary Fig. S3).

Finally, we also established the optimal freezing condition for the stable-transfected cell line for long-term storage. In particular, we compared three freezing conditions: (i) serum:complete medium (CM) (1:1) with 5% DMSO, (ii) CM with 5% DMSO, and (iii) serum only with 5% DMSO. As shown in Supplementary Fig. S4, we observed a higher cell viability after cell thawing when using both serum and complete medium (1:1) and a lower concentration of DMSO (5%). Higher amount of DMSO (10%) was toxic for the cells (image not shown).

### cpt-cAMP-mediated translocation of AQP5

**Figure 5:**
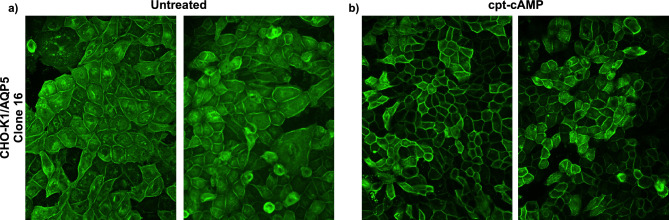
Effect of cpt-cAMP on the sub-cellular distribution of AQP5 in CHO-K1/AQP5 cells. Representative immunofluorescence images showing the distribution of AQP5 in (**a**) untreated and (**b**) 24 h cpt-cAMP treated CHO-K1/AQP5 cells. Magnification: ×40.

To address whether AQP5 translocation to the plasma membrane could be induced in our stable-transfected cell line, the CHO-K1/AQP5 cells (clone 16) were stimulated with chlorophenylthio-cAMP (cpt-cAMP) that is known to trigger AQP5 translocation in a murine lung epithelial cell line^3^. CHO-K1/AQP5 cells were treated with 200 µM cpt-cAMP for 24 h, and the immunofluorescence staining was performed. Cells treated with cpt-cAMP exhibited a stronger staining for AQP5 on the plasma membrane (Fig. 5b), showing that AQP5 can translocate to the cell membrane after stimulation with cpt-cAMP. As expected, unstimulated CHO-K1/AQP5 showed a visible AQP5 staining at the plasma membrane but less strong compared to cells stimulated with cpt-cAMP but also an AQP5 intracellular staining that was less visible for the cpt-cAMP stimulated cells (Fig. 5).

## Discussion

Aquaporin-5 (AQP5) is a water channel involved in the transport of water. The human protein is expressed in several tissues including salivary, lacrimal and sweat glands, as well as in stomach, pancreas and lung^10^. AQP5 monomer is formed by six transmembrane helices linked with three extracellular and two intracellular loops. The functional AQP5 is a tetramer composed by four identical monomers^2,10,11^. Anti-AQP5 autoantibodies in Sjögren’s syndrome (SS) target the three extracellular loops probably affecting the water flux^6^. SS patients with circulating anti-AQP5 antibodies have more severe sicca symptoms, suggesting a potential pathogenic role of these antibodies^12^. Therefore, screening for anti-AQP5 autoantibodies in SS patients might help to define a subset of patients and introduce targeted therapies. Moreover, a therapeutic approach targeting AQP5 might help to restore a proper water flux. In this scenario, developing monoclonal antibodies against AQP5 extracellular loops will potentially open the way for the development of new diagnostics and therapies in SS. AQP5 is a transmembrane protein, thus the expression in its native form as soluble recombinant protein is very difficult to achieve. Therefore, for phage display biopanning experiments, the use of whole cells as source of antigen could potentially overcome this problem. The MLE-12 murine lung epithelial cell line is a well-characterized model for membrane protein expression, including AQP5^3^. Though, to our knowledge, no human AQP5 stable cell line is currently available. Thus, this study was performed to generate a new cell line stably overexpressing human AQP5 as tool to produce recombinant monoclonal antibodies targeting AQP5 by cell-based phage display biopanning and potentially to study the molecular mechanism of AQP5 sub-cellular distribution.

In this work, we were able to generate a stable-transfected CHO-K1/AQP5 cell line by transfecting CHO-K1 cells with a commercially available human AQP5 plasmid. We characterised one clone, named clone 16, that was able to stably express AQP5 h on the plasma membrane and also intracellularly over time by means of immunofluorescence and Western blot analysis. As expected, AQP5 was distributed both in the cytoplasm and in the plasma membrane similarly to its natural distribution whereby AQP5 is able to translocate from intracellular reservoirs to the membrane.

Moreover, we established the best cryopreservation medium for long-term storage of the CHO-K1/AQP5 cell line. For monoclonal antibody production, one of the advantages in using the CHO-K1/AQP5 cell line over the MLE-12 is also the availability of a control negative cell line (CHO-K1) not expressing AQP5 at any level, as we showed by immunofluorescence and western blot analysis.

Some membrane proteins can traffic from cellular reservoirs to the plasma membrane in response to proper stimuli. It has been demonstrated that cAMP can induce AQP5 translocation to the plasma membrane^3^. Therefore, we tested the effect of cAMP on our CHO-K1/AQP5 cells (clone 16). Unstimulated CHO-K1/AQP5 cells show a clear expression of AQP5 on the plasma membrane but also intracellularly. We showed that treatment with cAMP could significantly increase the translocation of AQP5 on the plasma membrane indicating that this stimulus can trigger AQP5 translocation in our stable cell line. This observation might be relevant for using our cell line not only for cell-based biopanning experiments but to perform functional studies on molecular mechanisms regulating AQP5 sub-cellular translocation.

**Figure 6:**
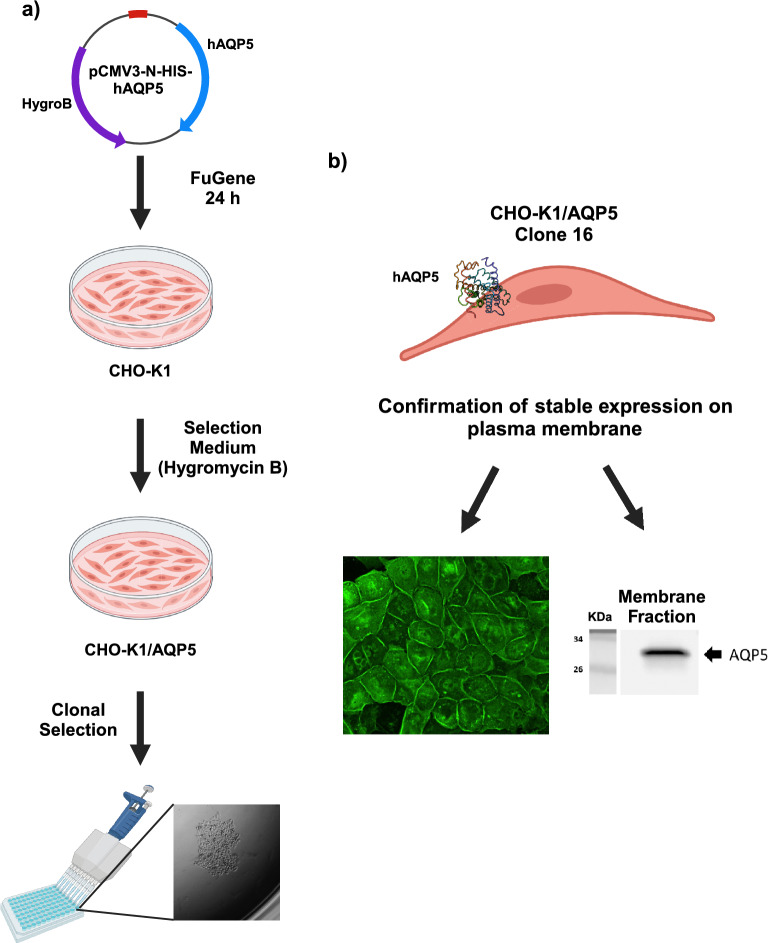
Generation and characterization of a CHO-K1/AQP5 human cell line. Graphical abstract summarizing all the steps performed to generate (**a**) and characterize (**b**) the CHO-K1/AQP5 cell line (clone 16). Image was prepared using BioRender (publication licence: QO26KQAHMZ).

In summary, we generated a new and efficient human CHO-K1/AQP5 cell line that could be used as tool to perform cell-based biopanning for the generation of monoclonal antibodies targeting AQP5 and to perform functional studies on AQP5 translocation (Fig. 6). In particular, the generation of antibodies displaying agonist activity towards AQP5 might be used as possible new therapeutics to restore exocrine function and salivary secretion for clinical use in SS.

## Materials and methods

### Cell culture

The CHO-K1 cells (subclones of the parental CHO cells; ATCC—CCL-61TM) were cultured in complete medium (CM) (HAM’s F12 medium (Gibco) supplemented with 10% fetal bovine serum (FBS) (Gibco) and 1% antibiotic–antimycotic (Gibco)) and kept at 37 ℃ with 5% CO_2_. The stably transfected CHO-K1/AQP5 cells were cultured in similar condition but using a selection medium (CM supplemented with 0.6 mg/ml of hygromycin B (Gibco)).

### Transfection of CHO-K1 cells with APQ5 plasmid

To generate CHO-K1/AQP5 cells, CHO-K1 cells were transfected with a pCMV3-N-HIS-hAQP5 plasmid (#HG14696-NH, Sino Biological) that carries a hygromycin B resistance gene for selection of mammalian cell lines. Briefly, CHO-K1 cells in CM were transfected using FuGene HD transfection reagent (Promega) following the manufacturer’s instructions. 24 h after transfection, medium was replaced with selection medium to select the stably transfected cells. To obtain clone populations derived from single cells, 10 days after selection, cells were detached with trypsin and dispensed in 96-well plates in a dilution of one cell per well in selection medium. After we obtained clone populations, cells on every well were trypsinized and expanded, and checked for hAQP5 expression by immunofluorescence.

### AQP5 detection by immunofluorescence

CHO-K1 and CHO-K1/AQP5 cells were plated onto 48-well plates or multi-spot glass slides for 24 h and fixed in acetone:methanol (1:1) for 30 min at − 20 ℃. After washing in 1× PBS and blocking with serum-free protein block (DAKO) for 1 h at room temperature (RT), cells were incubated with a rabbit monoclonal anti-human AQP5 antibody (Abcam) in antibody diluent (DAKO) for 1 h at RT. After washing with 1× PBS, Alexa Fluor-488 donkey anti-rabbit IgG (Invitrogen) was applied for 1 h at RT. 4ʹ, 6- diamidino-2-phenylindole (DAPI) (Invitrogen) was added to visualize the nuclei. 48-well plates and slides were imaged using an inverted fluorescence microscope Leica DMi8 or Nikon spinning disk confocal microscope for better visualization of the membranes or the Olympus BX60 microscope.

### AQP5 detection by western blot

CHO-K1 and CHO-K1/AQP5 cells were washed twice with cold 1× PBS and lysed in RIPA buffer (Thermo Scientific) with protease inhibitor cocktail (Sigma). After 1 h on ice, samples were centrifuged at 20,000×*g* for 10 min at 4 °C. Supernatants were recovered and the protein concentration was measured using BCA protein assay kit following the manufacturer’s instructions (Thermo Scientific). protein extract (5 µg) was resolved using SDS–PAGE and transferred to a nitrocellulose membrane (GE Healthcare Life Science). The blocking was performed in 5% (W/V) non-fat dry milk in TBST (TBS plus 0.1% Tween-20). AQP5 expression was detected using a rabbit monoclonal anti-AQP5 (Abcam), followed by HRP-conjugated goat anti-rabbit IgG (Abcam). Immunoreactivity was assessed by a chemiluminescence reaction using Clarity Western ECL substrate (BioRad) and visualized in Gel documentation system (Syngene G:BOX).

### Membrane protein detection

Cell membrane proteins were biotinylated with EZ-Link^™^ Sulfo-NHS-Biotin (Thermo Scientific)^13^. Briefly, CHO-K1 and CHO-K1/AQP5 cells were plated in culture-treated petri dishes for 24 h. After washing with ice-cold 1× PBS supplemented with CaCl_2_/MgCl_2_ (PBS+/+), cells were incubated for 30 min at 4 ℃ with 0.5 mg/ml of Sulfo-NHS-SS-Biotin prepared in PBS+/+ to biotinylate the extracellular part of the membrane proteins. After washing with biotin quenching solution (100 mM of glycine in PBS+/+), cells were lysed in RIPA buffer (supplemented with protease inhibitor cocktail). The total protein lysate was incubated with streptavidin agarose resin beads (Thermo Scientific) for 2 h at 4 ℃ in RIPA buffer. The unbound supernatant corresponds to the non-membrane fraction. Beads were washed 3 times with RIPA buffer and eluted adding 1× Laemmli buffer (membrane fraction). Samples were boiled at 70 ℃ for 7 min and loaded for western blot analysis to detect AQP5 and β-actin (monoclonal anti-β-actin-peroxidase from Sigma–Aldrich)) as a *fractionation* control.

### Detection of HIS tag on AQP5 by ELISA

Sandwich ELISA assay was performed to detect the N-terminal HIS tag on AQP5. Briefly, NUNC Maxisorp 96-well Immuno plates (Thermo Scientific) were coated overnight at 4 **℃** with 2 µg/ml of capture antibody anti-AQP5 (Abcam) in 1× PBS, followed by blocking with 3% BSA/1× PBS for 2 h at RT. After washing with 1× PBS, 50 µg/ml of CHO-K1 or CHO-K1/AQP5 protein extracts were added to the wells in blocking solution, and incubated for 2 h at RT. After another round of washes with 1× PBS, two alternative detection systems were used in parallel: (1) anti-HIS-HRP antibody (one in 5000) (Abcam) or (b) HisProbe-HRP (2 µg/ml) (Thermo Scientific), both in 3% BSA/1× PBS and incubated for 1 h at RT. After washing, the assays were developed using tetramethylbenzidine (TMB) substrate reagent set (BD Bioscience). Optical densities (ODs) were measured at 450 nm.

### cpt-cAMP treatment

CHO-K1/AQP5 cells were seeded onto multi-spot glass slides. Next day, cells were starved for 24 h with starvation medium (HAM’s F12 medium supplemented with 1% FBS, 1% antibiotic–antimycotic and 0.6 mg/ml of hygromycin B). The day after, medium was removed, and cells were treated for 24 h with 200 µm of 8-cpt-cAMP (Santa Cruz Biotechnology) or vehicle (bdH_2_O) in CM. The subcellular localisation of AQP5 protein was determined by immunofluorescence (described above) followed by visualization on a Nikon spinning disk confocal microscope.

### Supplementary Information

Supplementary Figure S1.

Supplementary Figure S2.

Supplementary Figure S3.

Supplementary Figure S4.

Supplementary Figures.

Supplementary Information.

## Data Availability

All data is included in this article.
