# Supplementary figures and images for: Efficient generation of a stable CHO-K1 cell line overexpressing the human water channel aquaporin-5 as tool to generate therapeutic antibodies
Source: Sci Rep. 2024 Jul 10;14:15992. doi: 10.1038/s41598-024-67147-x (PMC11237030; doi:10.1038/s41598-024-67147-x)

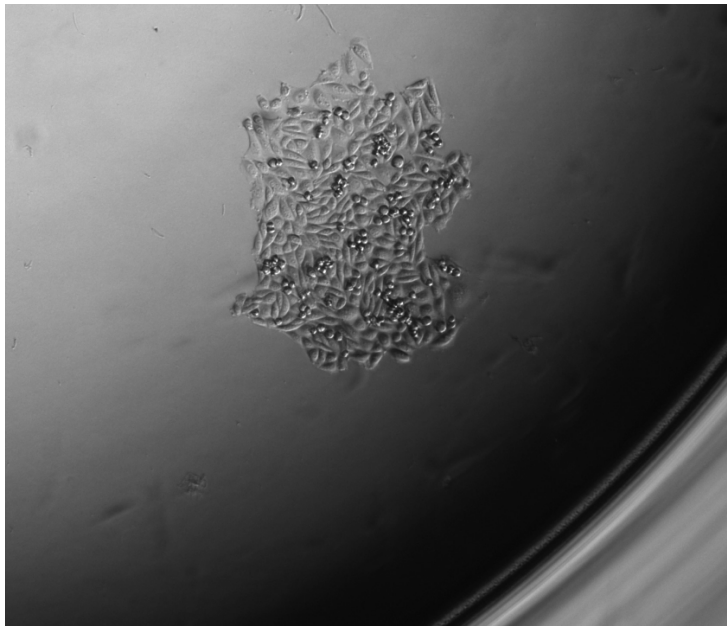

Supplement: Supplementary file 1 — Supplementary Figure S1. [file 41598_2024_67147_MOESM1_ESM.pdf]

**Clone 16**

**DAPI**

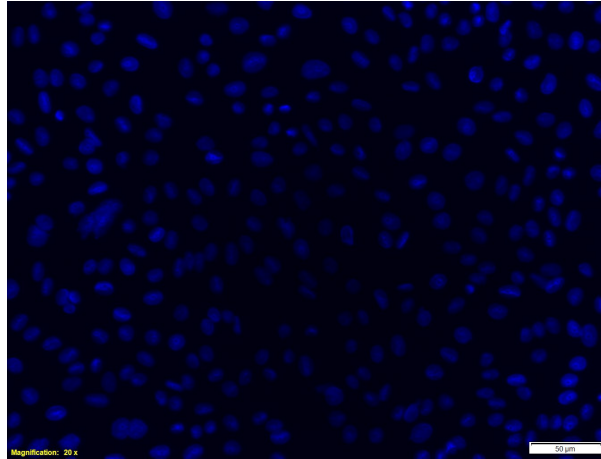

**APQ5**

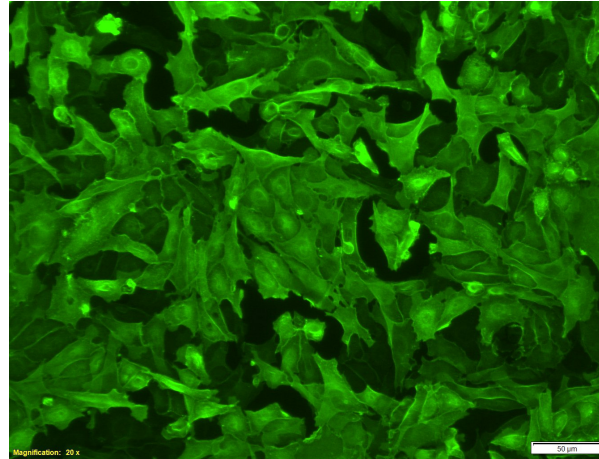

**Merged**

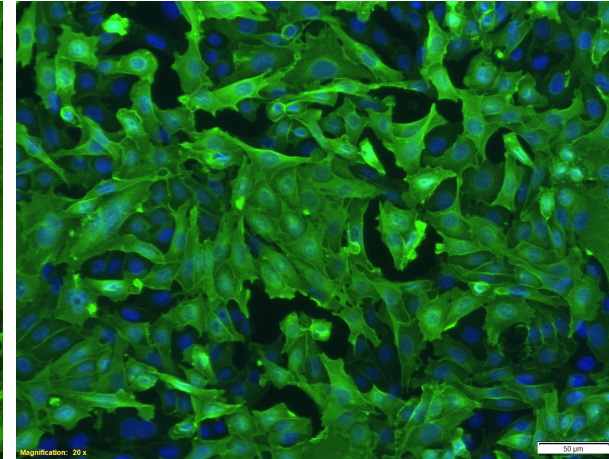

Supplement: Supplementary file 2 — Supplementary Figure S2. [file 41598_2024_67147_MOESM2_ESM.pdf]

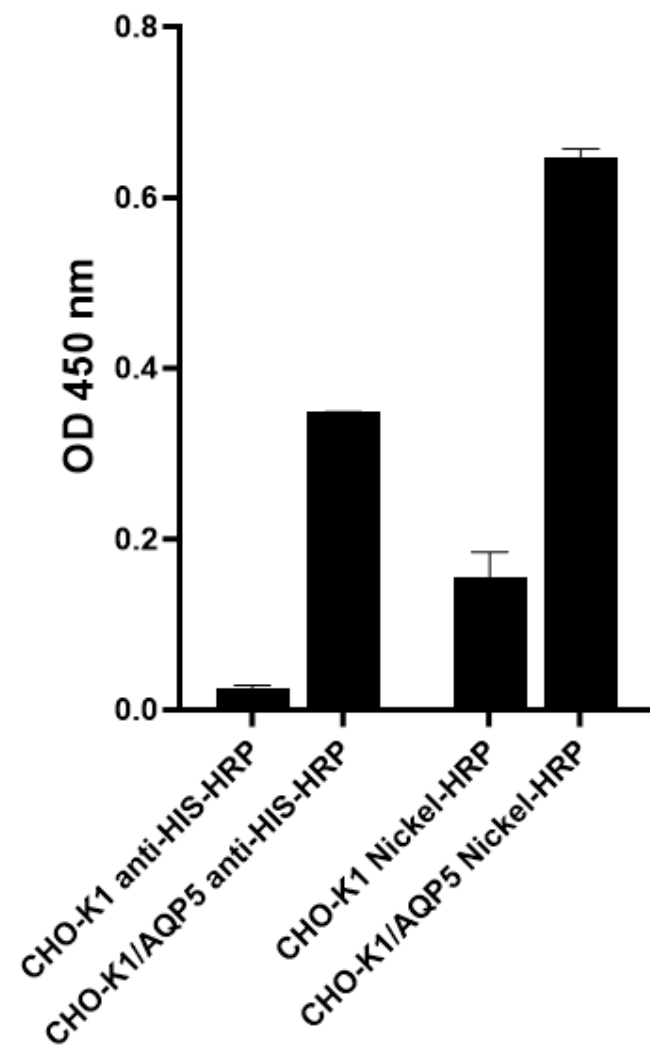

Supplement: Supplementary file 3 — Supplementary Figure S3. [file 41598_2024_67147_MOESM3_ESM.pdf]

**Serum: CM (1:1) + 5% DMSO**

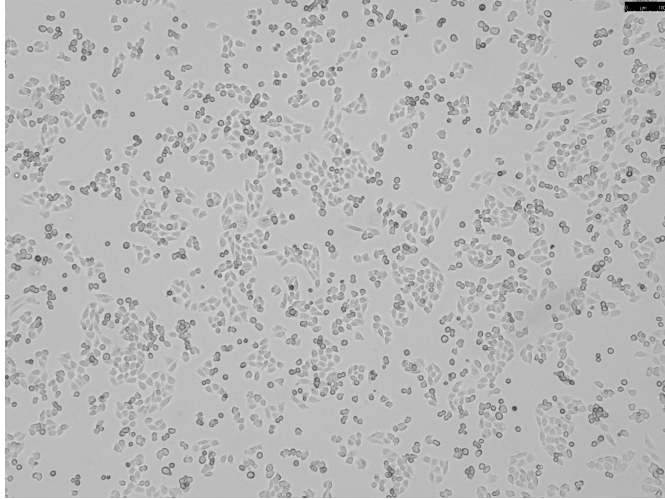

**CM + 5% DMSO**

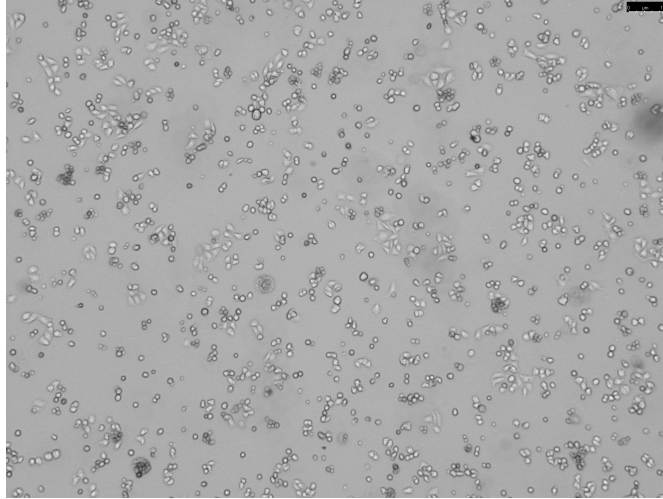

**Serum + 5% DMSO**

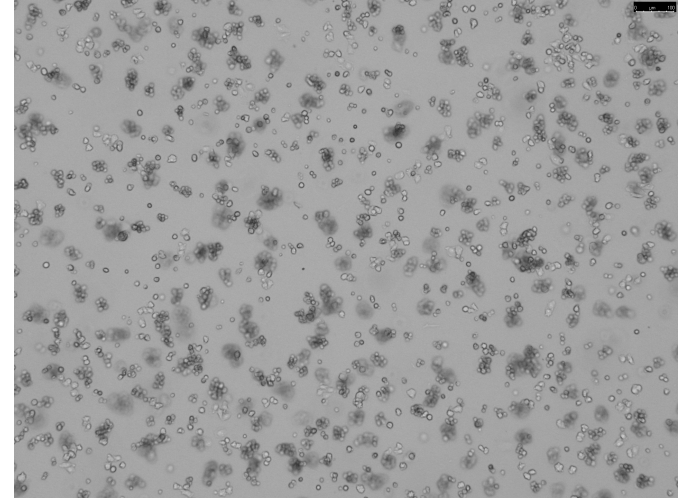

Supplement: Supplementary file 4 — Supplementary Figure S4. [file 41598_2024_67147_MOESM4_ESM.pdf]

Figure S5

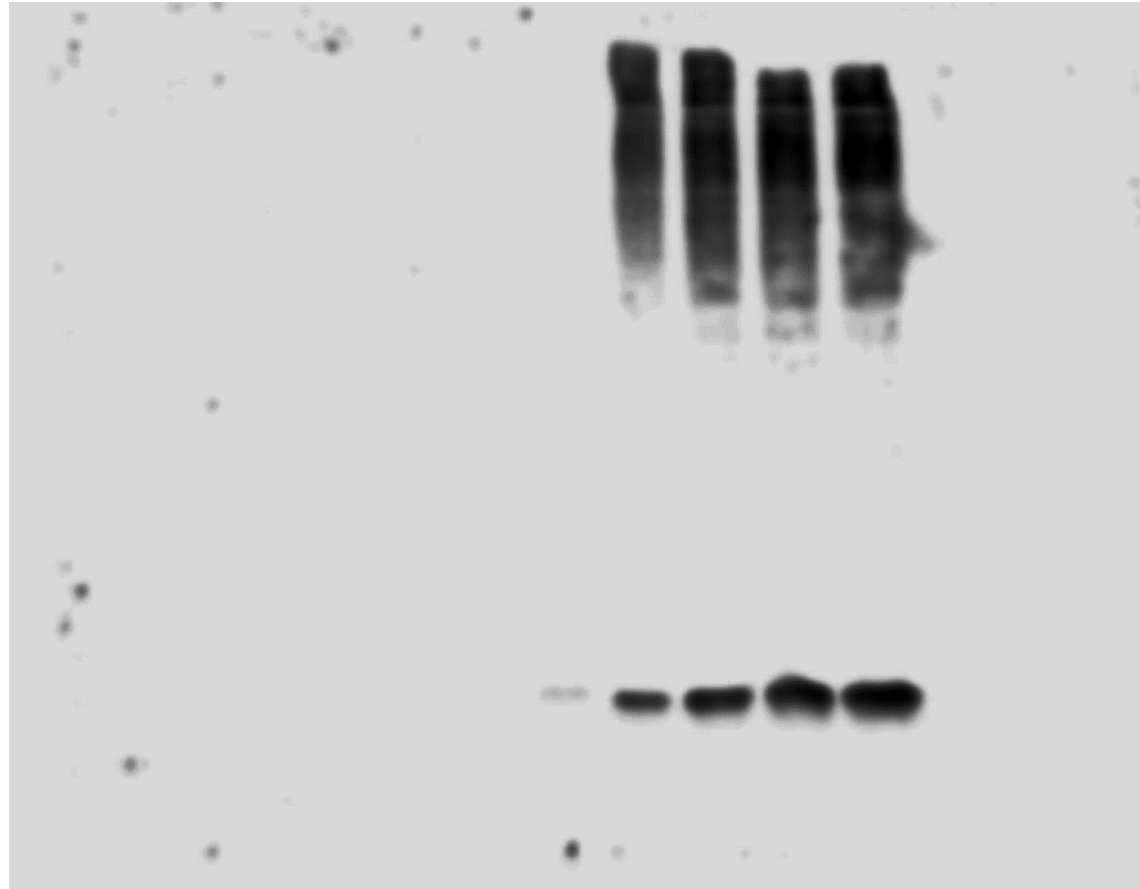

Figure S6

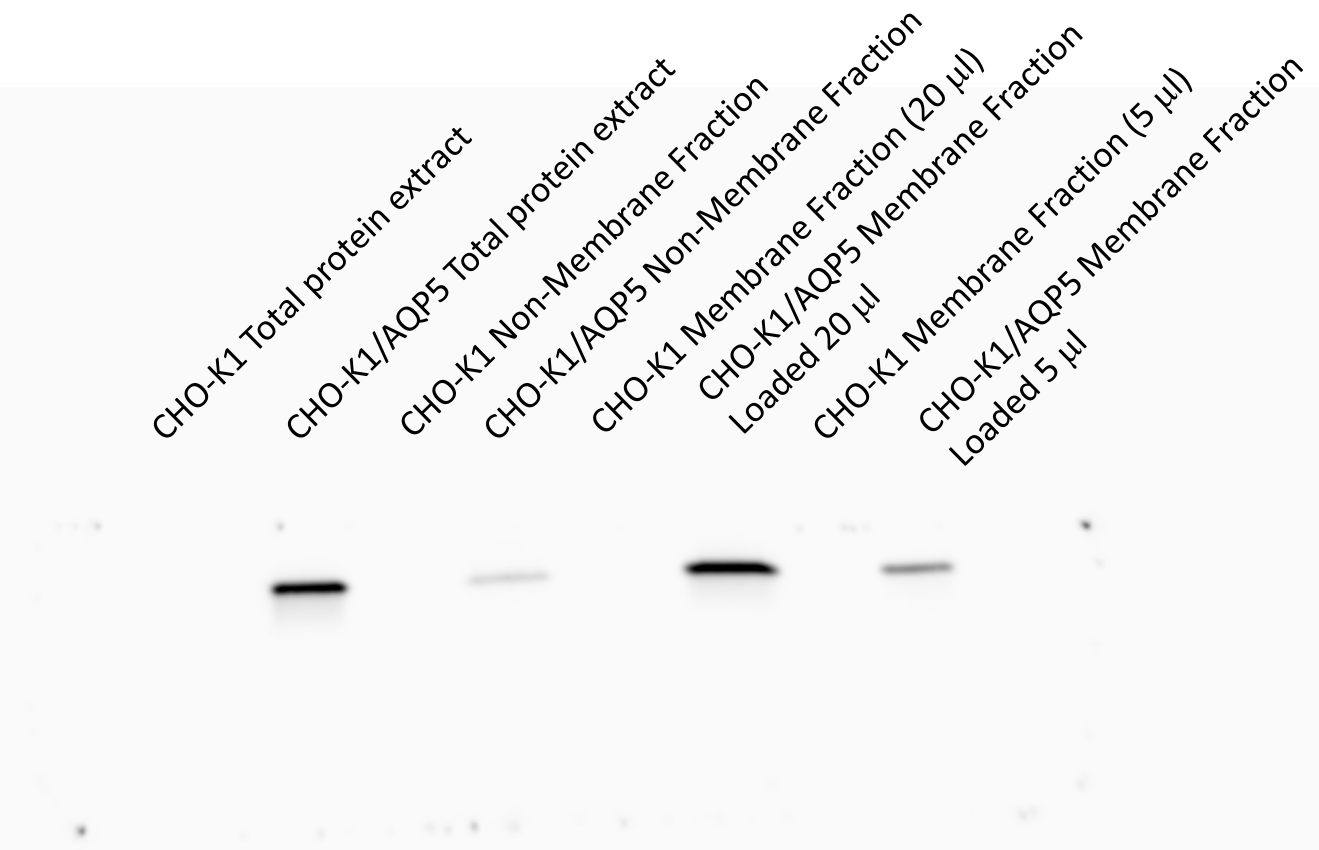

Figure S7

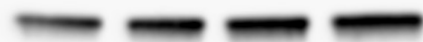

Supplement: Supplementary file 5 — Supplementary Figures. [file 41598_2024_67147_MOESM5_ESM.pdf]
