# Supplementary material for: Efficient generation of a stable CHO-K1 cell line overexpressing the human water channel aquaporin-5 as tool to generate therapeutic antibodies
Source: Sci Rep. 2024 Jul 10;14:15992. doi: 10.1038/s41598-024-67147-x (PMC11237030; doi:10.1038/s41598-024-67147-x)
Supplement: Supplementary file 6 — Supplementary Information. [file 41598_2024_67147_MOESM6_ESM.docx]

**Supplementary Figure Legends**

**Supplementary Figure S1. CHO-K1/AQP5 clonal selection.** Representative bright field image of one well of a 96-well plate in which the single cells dilution was performed. Image shows cell aggregates derived from one single cell.

**Supplementary Figure S2. CHO-K1/AQP5 clone 16.** Representative immunofluorescence pictures showing staining for AQP5 (green) for clone 16. Nuclei were stained with DAPI (blue). All images have been acquired using the Olympus BX60 microscope. Magnification: 20X.

**Supplementary Figure S3.** **Detection of HIS-tag in the N-terminal of AQP5.** ELISA test results showing the HIS-tag detection on protein extract from CHO-K1 and CHO-K1/AQP5 cells using either an anti-HIS-HRP antibody or Nickel-HRP probe. Results are expressed as absorbance at 450 nm.

**Supplementary Figure S4. Cryopreservation test for CHO-K1/AQP5.** Representative bright field images of CHO-K1/AQP5 cells after thawing from three different freeze media: i) serum:complete medium (1:1) + 5% DMSO (left), ii) complete medium + 5% DMSO (middle), and iii) serum + 5% DMSO (right).

**Supplementary Figure S5.** Original image for Figure 4a is displayed.

**Supplementary Figure S6.** AQP5 expression on non-membrane and membrane fraction from CHO-K1 and CHO-K1/AQP5 cells (clone 16) assessed by Western blot. Original image is displayed. For the membrane fraction, two loading concentrations were used: 20 μl and 5 μl.

**Supplementary Figure S7.** β-actin original image for Figure 4b is displayed.
